# High-resolution ptychographic nanoimaging under high pressure with X-ray beam scanning

**DOI:** 10.1073/pnas.2514163122

**Published:** 2025-10-24

**Authors:** Tang Li, Ken Vidar Falch, Jan Garrevoet, Leonid Dubrovinsky, Mikhail Lyubomirskiy

**Affiliations:** ^a^Centre for X-ray and Nano Science, Deutsches Elektronen-Synchrotron, Hamburg 22607; ^b^Photon Science, Deutsches Elektronen-Synchrotron, Hamburg 22607, Germany; ^c^Bayerisches Geoinstitut, University Bayreuth, Bayreuth D-95440, Germany; ^d^Science Division, MAX IV Laboratory, Lund University, Lund 221 00, Sweden

**Keywords:** X-ray microscopy, high pressure, lens-less imaging, phase-contrast

## Abstract

We present an approach to nanoscale-resolution high-sensitivity imaging of internal material structure under in situ/operando conditions for virtually any sample environment. When bulky or heavy sample environment is required state-of-the-art X-ray imaging techniques, such as scanning and full-field microscopy or holography fail to deliver high-resolution imaging capabilities due to either i) extremely small optics’ working distance for magnification-based methods or ii) the inability to precisely control heavy sample position in the case of lens-less methods. In this work, we address those challenges for a scanning lens-less imaging method called ptychography. Instead of precisely controlling the sample position during raster scan in a focused, confined X-ray beam, we are scanning that beam across the sample. This overcomes the constraints on scanning procedure imposed by sample size/weight and delivers unmatched scanning speed while maintaining high precision of beam position during the scan. We directly applied our approach, showcasing phase contrast nanoimaging with diamond anvil cells, and visualized intricate details of the melting and oxidation of laser-irradiated iron under pressure of 50 GPa.

## Significance

We demonstrate a novel approach to X-ray ptychography, a phase-sensitive scanning microscopy method. By replacing the conventional sample scanning approach with a beam-scanning via reflective optics, we expand the capabilities of nanoimaging for heavy/bulky sample environments, e.g., under extreme pressure. Our approach eliminates the major limitation of previous methods: the necessity to translate the sample. This long hindered high-resolution imaging in environments such as diamond anvil cells. By steering the X-ray beam, we enabled visualization of pressure-driven reactions (e.g., iron oxidation at 50 GPa) with the highest sensitivity and sub-50 nm resolution. This breakthrough opens an avenue for operando nanoscopy, benefiting many scientific fields, such as geoscience, materials synthesis, and high-pressure physics, where static imaging methods fall short.

Microscopy has been a driving force behind human development for over 400 y. Visible-light microscopy is still widely used in various distant disciplines, from microbiology and medicine to geoscience. However, conventional optical microscopes are limited in providing resolution beyond a submicrometer. On the other hand, electron microscopy, such as scanning and transmission, experienced spectacular development and now can provide a resolution of up to 0.5 Å (1). Unfortunately, due to the small penetration depth in most materials, electron microscopy provides only surface information in most cases or requires the preparation of extremely thin sample slices, restricting functional studies.

X-ray microscopy can achieve a resolution that falls between visible light and electron microscopy. Compared to visible light and electrons, X-rays have a longer penetration depth, which makes them an attractive option for nondestructive nanoimaging of internal material structure. This opens up possibilities for functional studies (in-situ/operando). The best resolution with X-ray microscopes can be achieved at synchrotron radiation facilities (SRFs), which provide spectacular peak brilliance. Generally, there are two approaches to X-ray microscopy, optics-based and lens-less; both, are capable of reaching single-digit nanometer resolution (2–4).

In for any type of microscopy the ultimate resolution is limited by the imaging system numerical aperture (NA) and wavelength of radiation. In optics-based microscopy the resolution is limited by the optics NA and its manufacturing quality. In such schemes the sample is either placed near the front or back focal plane of the focusing optics and pixel detector records the magnified sample image (full-field scheme), or 0-D detector records intensity while sample is being scanned by a diffraction limited beam (scanning probe scheme). Despite wide usage, these schemes have limitations.

The requirements for manufacturing high-resolution optics, particularly for X-ray energies above 10 keV) present significant challenges. Creating optics with a large NA (up to tens of milliradians) and a long focal distance necessitates precise control in manufacturing process, with subnanometer level across hundreds of micrometers for diffractive optics, and single-digit nanometers over distances of tens of centimeters for total reflective optics. This leads to significantly reduced optics working distance, as the sample needs to be placed in close vicinity of the focal plane. This constrains working distance of large NA optics to few centimeters, e.g. in case of Multilayer Laue Lenses it is even less than 2 mm (4) or for high-resolution K-B mirrors, known for their largest working distance, it does not exceed 10 cm (5–8). Even with energies below 10 keV 5 cm is considered to be a “large” working distance (9). This is a major limitation for performing high-resolution optics-based microscopy in situ or operando because the sample environment chamber does not fit between the optics and the focal plane.

However, lens-less imaging techniques, such as coherent diffraction imaging (10, 11) and its scanning counterpart—X-ray ptychography (12) are able to achieve resolutions in the single-digit nanometer range (13, 14) without the need for large NA focusing optics. In these methods, the combination of a 2D detector and the sample acts as a large NA imaging system. The detector serves as a “virtual” lens, directly recording the modulus of the object’s Fourier transform. In these approaches, the ultimate resolution is determined by the scattering signal produced by the sample.

In hard X-ray ptychography, a confined coherent beam is used to irradiate the sample at various positions. A 2D detector records the diffraction signal from the sample at each of these scanning positions. By precisely knowing the beam’s position on the sample and the corresponding recorded diffraction signals, it is possible to iteratively reconstruct both the sample’s complex transmission function and the probing beam. Being a lens-less imaging technique, ptychography can use focusing optics to enhance photon fluence on the sample, thereby accelerating data collection.

In the last decade, X-ray ptychography has gained significant popularity as a method for nondestructive visualization of internal structures of many ex-situ sample systems (15–18) and has started to expand into the field of functional and in-situ studies (19, 20).

Currently, two major challenges hold back the widespread application of hard X-ray ptychography for situ and operando measurements: i) low number of coherent photons in hard X-ray regime (above 10 keV) limits studies of thick samples, and ii) nanometer control and stability of sample positions during rapid scanning in a heavy/bulky in-situ/operando cell required for maintaining high resolution.

Lack of coherent photons can be addressed either by the upgrade of SRFs to fourth generation (21, 22), which reduces electron beam emittance increasing number of coherent photons in the X-ray beam (and also making it less divergent), or reusing incoherent photons in parallel scanning scheme to speed-up data acquisition procedures (23–26).

However, the problem of precise and stable positioning of heavy and/or bulky samples (together with their environment) is much more severe than it seems; it often acts as a show stopper for performing ptychography, for example for high-pressure physics and chemistry, which represent a rapidly developing interdisciplinary field focused on the synthesis of new materials and the study of their responses to applied pressure. Such conditions can alter a material’s structural, electronic, and magnetic properties. High static pressures are achieved using various devices, ranging from large-volume presses (up to several tons of weight) to relatively compact diamond anvil cells (DACs). Among these, DACs are unparalleled, enabling pressures up to 1 TPa (27).

A new scanning approach is essential to enable high-resolution X-ray imaging for in-situ/operando conditions in general and DAC in particular. If moving the sample is associated with prohibitive challenges, unleashing the beam and allowing its free move across the sample is the remaining solution; PSI (28) and APS(29) have performed efforts toward the realization of such an approach with X-rays using translation scanning of Fresnel zone plate (FZP) for speeding up ptychography data acquisition at third generation synchrotrons and softer X-rays. Unfortunately, such an approach cannot address all challenges, especially in the case of fourth generation SRFs where the coherence fraction of the beam is much higher, and thus, FZP will occupy a much larger portion of the incoming highly collimated beam, leaving no reasonable space for transverse scanning. Another drawback of such an approach is that it is only suited for FZP—a lightweight optics that is not sensitive to small angular variations; in the case of reflective or refractive optics, the problem with moving large weights will be accompanied by the need to maintain tight angular alignment if higher X-ray energies are required.

Thus, the only remaining option is to tilt reflective optics effectively moving the X-ray beam across the sample; such an approach has been demonstrated in visible light with so-called “galvo mirrors” (30–32). However, up until now, such a scheme has not been demonstrated for any imaging application with shorter wavelengths, especially with X-rays. This imaging scheme poses a number of challenges, such as variable aberrations introduced by imperfect tilting mirror (33), precise determination of the beam position on the sample, and variable position of the beam at the detector during the scan which disrupts the inverse problem solution.

In the present work, we overcome the above-mentioned challenges and demonstrated the practical realization of the concept of a ptychographic imaging experiment in which the sample is scanned by a fully coherent beam freely moved by tilting reflective optics. We applied this concept to enable the visualization of the intricate details of Fe melting in DAC under extreme conditions.

## Results

**Fig. 1.**
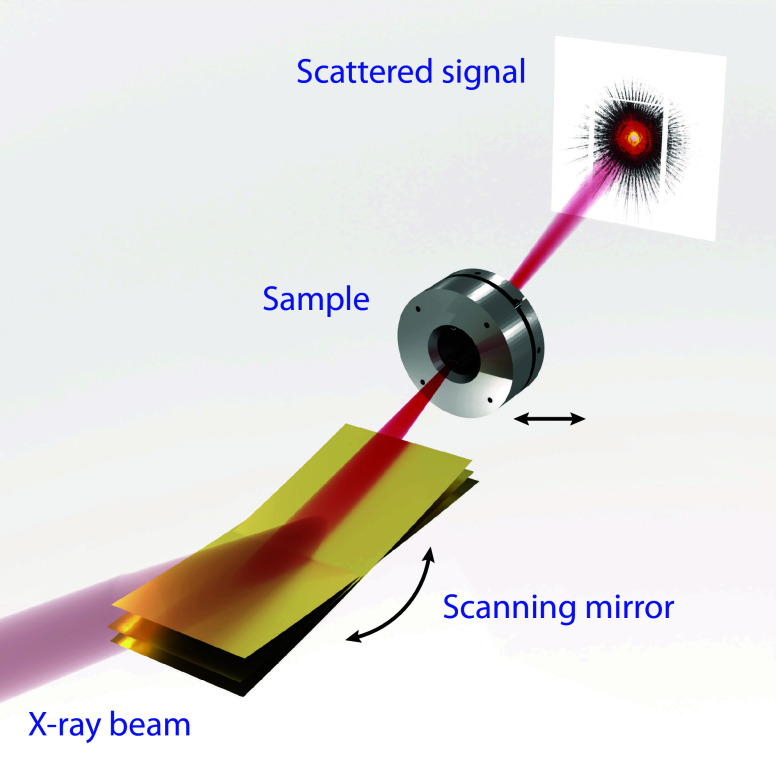
Scheme of the experimental set-up showing 3 out of many positions of the scanning mirror. Arrows indicate scanning movements of both, mirror and sample.

The experiments were carried out on the microprobe branch of the P06 beamline of PETRA III SRF. The experimental scheme is depicted in Fig. 1, the X-ray beam was focused by a set of Kirkpatrick Baez (KB) mirrors (not shown) with a working distance of 200 mm (last mirror edge to sample), the scanning mirror was placed between the last KB mirror and the sample, a beam cleaning aperture (not shown) was placed in the close vicinity of the sample to suppress background scattering. The samples were raster scanned with a scanning mirror oscillating in a vertical direction acting as a fast scanning axis and a motorized stage moving the sample in a horizontal direction acting as a slow axis. The Eiger detector was used to record diffraction patterns in the far field.

The beam’s position on the sample was retrieved from the data obtained by the set of interferometers tracking the movement of the scanning mirror and correlated with beam center positions at the far field detector obtained on “empty” scan. This approach has the highest precision for the determination of the position of the X-ray beam on the sample as it is performed by direct measurement of the mirror displacement during exposure with subnanometer precision.

### Set-Up Validation.

**Fig. 2.**
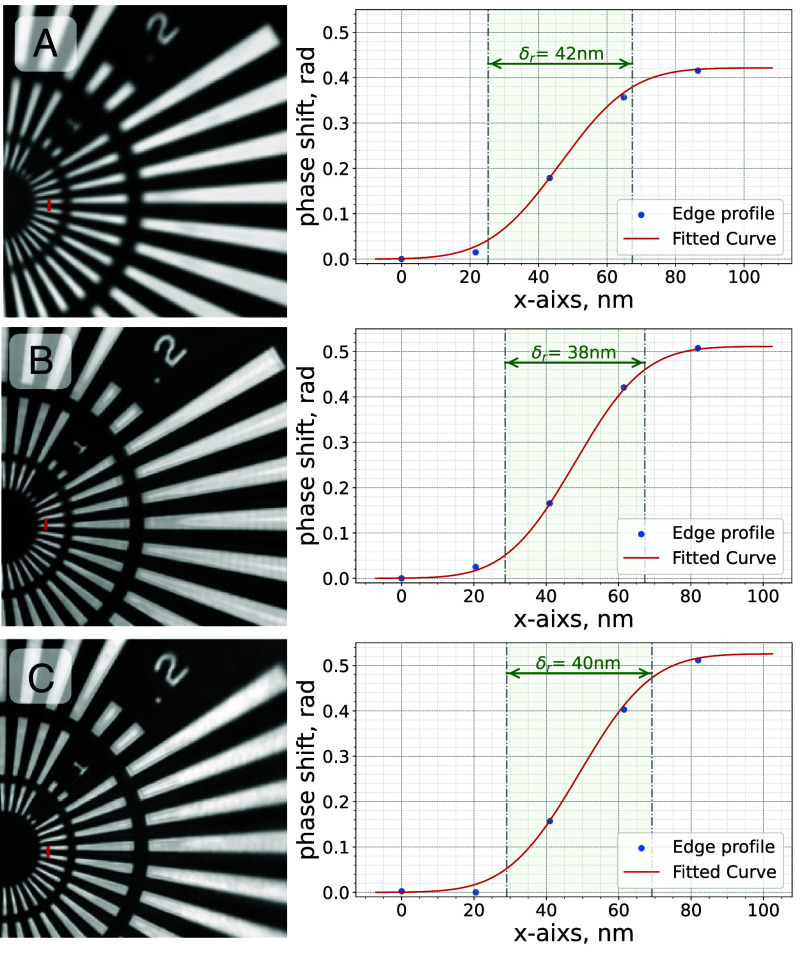
Comparison of the cropped reconstructed phase shift of the part of the scan of test structure—Siemens Star. The scanning was done using scanning mirror (*A*), stage continuous scan (*B*), and stage step scan (*C*). Fast scanning axis is vertical.

The new experimental scheme was first validated on a well-known test object Siemens Star (34), Fig. 2 shows the reconstructed phase shift of the beam tilting scan in comparison with conventional scanning schemes performed at the beamline: step scan and “fly” scan (constant sample movement). As the commissioning result, we achieved the photon statistics limited resolution of 42 nm (edge response) and 36 nm (FRC, 1 bit criterion, see *SI Appendix*) with mirror scan (12.3 keV X-rays). The average photon count per diffraction pattern was approximately $1.65\times 10^{6}$. The achieved resolution (edge response) of the reconstructed image with a mirror scan is comparable to 38 nm resolution (edge response) obtained with the regular sample “fly” scan (13 keV X-rays) and the 40 nm resolution (edge response) step scan (13 keV X-rays). Additionally, we conducted FRC analysis on two conventional scans (step and fly) in the same scanning region, resulting in a resolution of 30 nm, which is again comparable to that of the mirror scan. These reference scans had photon counts of approximately $1.03\times 10^{7}$ photons per diffraction pattern, which is 6.3 times more compared to the mirror scan. The translation stage laser encoder recorded the positions of the sample during scans. All of the scans were taken with identical steps or integration intervals in order to sample the same frequencies of possible sample-beam vibrations.

The difference in total flux observed per diffraction pattern between mirror tilting scans and sample translating scans arises from the challenges of performing both types of scans in a short time interval. This difficulty caused by the need to dismantle the mirror scanning setup. To eliminate any potential influence of the mirror on the translational scans, such as additional angular vibrations, the mirror tilting setup had to be completely removed. This process resulted in a significant time delay of more than one day which affected overall logistics of the experiment due to potential conflict with another experiment and subsequently necessitated realignment of the beamline optical scheme.

On the other hand, to maintain the same detector integration time and thereby capture the same mechanical vibration frequencies, we made the decision to compromise on spatial resolution for the mirror scans. We focused on conducting comparative measurements that favored the translational scans.

### Imaging Under High Pressure: Fe Oxidation.

A BX-90 DAC equipped with Boehler-Almax diamonds featuring flat culets measuring 250 μm, affixed to tungsten carbide seats, was utilized for pressure generation. A Re gasket, preindented to a thickness of approximately 14 μm, was drilled to create a sample chamber with a diameter of about 110 μm. A thin piece of Fe foil, approximately 20 μm in lateral dimensions and 3 μm thick, was placed inside the sample chamber to react with O2. The cell was cryogenically loaded with O2, serving as a chemical reactant and pressure-transmitting medium. The pressure was determined to be 51(2) GPa before laser heating, based on the equation of the state of hexagonal close-packed Fe. The foil was heated using a double-sided near-infrared laser at the Bayerisches Geoinstitut to a temperature of 2,350(150) K for 2 s, with the laser beam focused to approximately 7 μm full width at half maximum (FWHM). X-ray single-crystal analysis of the reaction zone revealed the formation of cubic FeO2 (space group Pa$\bar{3}$, lattice parameter a = 4.3869(9) Å (35). Afterward, the DAC was shipped to DESY and scanned using our approach at a Siemens Star validated setup with 13 keV X-rays.

**Fig. 3.**
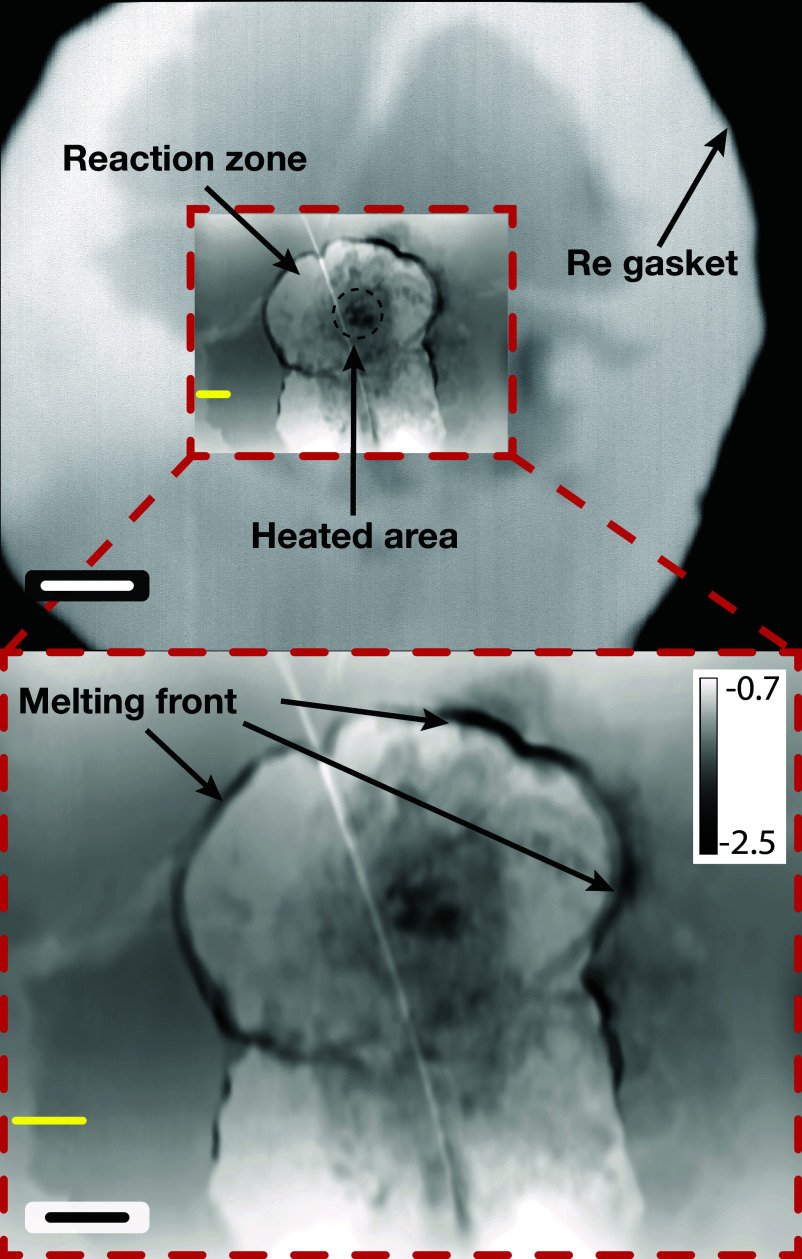
The image of the content of DAC. *Top*–total transmission image of the whole Re window with insert of the reconstructed phase shift; *Bottom*–enlarged image of central part reconstructed using the ptychography method. (Black scale bar represents 3 μm the white one to 10 μm, the phase shift is in radians.) The pixel size is 41 nm. Yellow bar indicates the place where absorption and phase shift of the Fe foil was measured.

The reconstructed phase shift image (Fig. 3) identifies the laser-heated area and shows that the reaction zone of approximately 27 μm in height and 18 μm in width is bigger than the actual laser beam size of 7 μm FWHM. It is also possible to distinguish between reacted and nonreacted zones on the foil by a melting front.

Moreover, the total area of the foil in the image is approximately 6 to 7 times larger than its area before the compression (20 μm diameter). Taking into account original thickness of the foil of 3 μm and assuming homogeneous thinning of compressed foil we estimate the amount of material in the beam corresponding to a thickness (with ambient conditions) in the range of 0.42 μm to 0.5 μm. Along with that we also identified reduced absorption of X-rays of nonreacted foil on the level of 3%, which corresponds to the amount of material of a thickness (with ambient conditions) of 0.5 μm. This value is in agreement with increased foil area and with foil phase shift of −0.23 rad measured on reconstructed ptychography image, this corresponds to the amount of material of a thickness (with ambient conditions) of 0.4 μm. The phase shift and absorption were measured on the same region of the image indicated with yellow bar—see Fig. 3.

Additionally, the high-resolution reconstructed phase image reveals that the reaction products are not homogeneously distributed within the laser-heated area. At least 3 distinct phases, presumably Fe, FeO2 and O2, can be distinguished by their differing phase shifts. A small Fe center is surrounded by a thick layer of FeO2, which in turn is surrounded by solid O2. On the outside of the O2 layer, there is a thin layer of increased Fe density, followed by FeO2. Additionally, it is easy to identify the size of individual grains of the reacted material to be sub- μm sized.

## Discussion

In our current work, we have pioneered an approach of scanning a sample with a tilting beam in X-ray ptychography, enabling nanoscale imaging of extreme physical conditions. Particular interest in our approach comes from high-pressure research, since high-resolution phase-contrast imaging in DACs is a new avenue. Several methods have reported detection of melting in laser-heated DACs, including optical observation, X-ray diffraction, X-ray absorption, and Raman spectroscopy (36–38). However, considerable difficulties in detection of melting under high pressures have led to significant inconsistencies in reported melting curves for the same materials (39, 40). Recent experiments on X-ray full-field transmission microscopy with an objective lens showed some potential for studying processes in DACs, e.g., melting (41). However, these experiments did not evolve beyond feasibility tests due to prohibitive challenges, such as poor spatial resolution (sub-μm), low sensitivity due to the ability to record only absorption contrast, which is not sufficient to reveal melting or chemical reactions that result in minimal changes of metallic density. On the other hand, the realization of high-resolution full-field X-ray holography is limited by the small working distance for both variants with the objective lens (2, 42) (working distance optics-sample is less than few mm) and projection scheme, e.g. with large NA optics ID 16A at ESRF working distance does not exceed 30 mm (5, 6), which makes it nearly impossible to accommodate DAC on the stage near the focus plane. The Fe-O system has garnered significant interest in high-pressure physics, geosciences, and related fields, yielding numerous exciting discoveries in the past decade (35, 43, 44). Experiments on the chemical interaction of pure Fe and O2 are particularly challenging because, above 5 GPa, solidified O2 becomes opaque, making optical observation of laser-heated samples (Fe embedded in O2 and reaction products) impossible.

Such detailed information about the content of DAC only became accessible with X-ray ptychography. Our results provide a pathway to visualize dynamic processes such as melting, chemical reactions, and sample flow at high pressures and temperatures. In the future, coupling laser-heating setups for DACs with X-ray ptychography could revolutionize these studies.

In this proof-of-principle experiment, we scanned the beam using only one mirror in the vertical direction. For the horizontal direction, the sample was translated using the conventional scanning stage (slow axis) as it was allowed by the setup. It should be noted that taking into account the beam footprint at the mirror, the distance between mirror and the last KB mirror can be shortened, creating additional space for a second scanning mirror for deflection of the beam in a horizontal direction, keeping the same working distance of the optical system, which will allow operation of the setup with a stationary sample.

This scanning approach to lens-less imaging has applications much broader than geoscience; in this scheme, the scanning speed is not linked with the sample size, making it suitable for many operando studies, such as the morphological study of catalysts (20, 45, 46) with actual operational conditions, which require a large-volume reactor. Another example is high-power electronics failure during operation; it requires exceptionally high scanning speed and a complete and functional device, which is impossible to match using conventional piezo scanning sample stages. FEL has a unique field of applications; the ability to quickly and precisely manipulate an X-ray beam in space opens up avenues for studying dynamic processes requiring moving objects, such as serial femtosecond crystallography. With this approach, it is now possible to track the very same particle being dropped in the FEL beam without requiring accumulating a large statistical ensemble of data, avoiding excessive postprocessing and selecting “good” diffraction patterns.

## Materials and Methods

### Experimental Set-Up.

The beam scanning was performed by the total reflective mirror, 35 nm Ru coated, with a figure error of 2 nm rms and total length of 40 mm, located on piezo tilting stage and positioned at a distance of 110 mm from the sample and 90 mm from the last KB mirror correspondingly. The beam incident angle at a mirror plane was 4.5 mrad (at the middle of the scanning range) which corresponded to the beam footprint of 13 mm (beam size is 60 μm). The X-ray beam size in the mirror scan experiment at the sample position (Siemens Star) was approximately 200 nm × 260 nm FWHM in horizontal and vertical directions, respectively (reconstructed with ptychography). For DAC, it was approximately 2800 nm × 2800 nm in both horizontal and vertical directions. The effective beam integration length on the sample was set to 96 nm for the mirror scan. The reference step and fly scans were taken with 100 nm step size or integration intervals.

For each scan point, we measured the angular relative position of the mirror with a laser interferometer. The beam positions at the sample plane were calculated using interferometer data and distance between the sample and the scanning mirror.

During the experiment the mirror was oscillating (full available period: 0 to 0.8 mrad-0), for each oscillation of a mirror a sample was translated by one step of 100 nm. Effective beam integration length on the sample was set to 96 nm and detector exposure time and mirror oscillation frequency was adjusted accordingly for all scans. A mirror scan was covering a field of view (FoV) of 47 μm for Siemens star and 94 μm for DAC scans correspondingly. The mirror commissioning scan with Siemens star (Fig. 2*A*) was taken with 12.3 keV X-rays, step and fly scans without mirror (conventional scanning with the stage) were taken with 13 keV X-rays.

The diffraction patterns were recorded by the in-vacuum Eiger (2,048 × 2,048 pix) detector with 75 μm pixel size. Upon collection the diffraction patterns were numerically recentered to compensate for the movement of the deflected beam on the detector. We used laser interferometer positions of the mirror during each detector exposure to determine the deflection angle and recalculated the shift of the beam at the detector plane and validated them with determined positions of the beam center on the “empty” scan (without sample in a beam).

The cumulative fluence on the sample was approximately $1.891\times 10^{8}$ photos/μm^2^ for the Siemens Star mirror scan and $1.156\times 10^{9}$ photons per μm^2^ for step and fly scans. These values were estimated from the total number of photons recorded by the detector over the full scan and corrected for transmission losses, namely 61% transmission through 1.5 m of air and 93 % transmission through the 200 nm Ta Siemens star structure for 12.3 keV, and 67.7% and 94% correspondingly for 13 keV. The effective scan range was calculated as the product of the total number of scan points and the step size, with an additional margin equal to the FWHM on either side to account for the beam profile.

### Beam Position Calibration Pipeline.

**Fig. 4.**
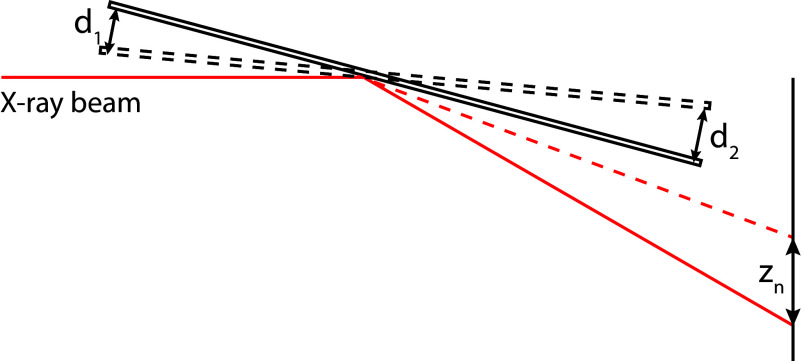
The schematic illustrates the mirror movement monitoring and scan range convention. As the mirror rotates by a small angle, it causes the interferometers to record the displacements $d_{1}$ and $d_{2}$, respectively. The lateral shift of the reflected beam at the sample plane is denoted by $z_{n}$.

To accurately determine the vertical beam position at the sample plane, the angular motion of a reflective mirror is monitored using two laser interferometers positioned at opposite ends of the mirror (Fig. 4) tracking displacement at its corresponding position. Two interferometers increase the angle determination precision as mirror’s pivot point is not known precisely, and the rotation occurs in the pitch direction, which is aligned with the beam axis.

Each interferometer records the displacement, $d_{1}$ and $d_{2}$, at a sampling rate of 12.2 kHz. To match the lower acquisition rate of the detector, we average these high-frequency measurements around the center of each detector integration interval, producing estimates of mirror’s angular motion. Those values are then used to compute the vertical beam displacement at the sample and detector planes.

#### 1) Laser displacement representation and mirror motion.

Each interferometer measures the displacement of the corresponding mirror point positioned on opposite sides of the mirror’s pivot point. To calculate the position of the beam at the sample plane, we use the scaling factors $\alpha_{1}$ and $\alpha_{2}$, which are preliminarily determined from a reference scan (without the sample) using the known geometry (mirror-sample-detector) and measured beam displacement at the detector plane. Using these factors, the displacement of the mirror surface measured with interferometers can is converted into vertical beam positions at the sample plane:

[1] $$\begin{array}{c} z_{1}=\alpha_{1}d_{1},\;z_{2}=-\alpha_{2}d_{2} \end{array}$$

Here, $z_{1}$ and $z_{2}$ represent independent estimates of the vertical beam positions at the sample plane, and $d_{1}$ and $d_{2}$ represent the mirror displacement recorded by interferometers.

#### 2) Improved beam position estimation.

Due to a lack of precise information on the distance from laser irradiated point on the surface of the mirror and its pivot point, the calculated beam positions $z_{1}$ and $z_{2}$ are not fully consistent with each other. To obtain more precise vertical positions of the beam at the sample and detector plane, the optimization of $\alpha_{1}$ and $\alpha_{2}$ is required:

[2] $$\begin{array}{c} \min_{\alpha_{1},\alpha_{2}}{\left|z_{1}-z_{2}\right|}^{2}={\left|\alpha_{1}d_{1}+\alpha_{2}d_{2}\right|}^{2} \end{array}$$

The final vertical beam positions at the sample plane is then obtained by averaging the positions $z_{1}$ and $z_{2}$.

Another aspect is the out-of-plane angular alignment. Since the detector coordinate system is not precisely aligned with the beam scanning axis, this misalignment leads to small horizontal shift of the beam on the detector, namely, the movement gains a diagonal component. Although this shift is relatively small (approximately 2 pixels), it needs to be accounted for the diffraction pattern recentering process. This shift was also estimated from the “empty” beam scan on the detector. To improve X-ray beam tracking accuracy, a third interferometer can be added.

### Ptychography Reconstructions.

Each diffraction pattern was recentered by converting the recalculated beam positions at the sample plane, horizontally and vertically, into corresponding pixel shifts on the detector. This correction provides possibility to process the ptychography data using conventional algorithms. Following this, ptychographic reconstructions were performed using a combination of algorithms: the ePIE algorithm (47) with position refinement (48) (every 50 iterations) for 5,000 iterations—Siemens star, 100 iterations—DAC, followed by the maximum likelihood (49) algorithm for an additional 5,000 iterations. The reconstruction process was performed using the open-source software package PtyPy (50). As we expected the tilting mirror to introduce variable aberrations; therefore, we limited the reconstructed vertical FoV to approximately 25 μm. Within this range, we observed no noticeable degradation in image quality. To further verify this, we performed a mixed-state reconstruction of the Siemens star test scan using four probe modes (51). In this reconstruction, we found that the dominant eigenmode accounts for 78 % of the total eigenvalue weight. We noticed little to no difference in reconstructed object quality between single and 4-mode reconstructions, see *SI Appendix* for more details.

## Supplementary Material

Appendix 01 (PDF)

## Data Availability

Raw data and recentering script have been deposited in Zenodo (DOI: 10.5281/zenodo.17121974) (52).
