# Supplementary material for: High-resolution ptychographic nanoimaging under high pressure with X-ray beam scanning
Source: Proc Natl Acad Sci U S A. 2025 Oct 24;122(43):e2514163122. doi: 10.1073/pnas.2514163122 (PMC12582291; doi:10.1073/pnas.2514163122)
Supplement: Supplementary file 1 — Appendix 01 (PDF) [file pnas.2514163122.sapp.pdf]

Supporting Information for

High-Resolution Ptychographic Nanoimaging under High Pressure with X-ray Beam Scanning.

Tang Li, Ken Vidar Falch, Jan Garrevoet, Leonid Dubrovinsky

Mikhail Lyubomirskiy
E-mail: mikhail.lyubomirskiy@maxiv.lu.se

This PDF file includes:

Supporting text
Figs. S1 to S3

Supporting Information Text

Probe Modes Comparison

To assess the influence of the reflective mirror on the probe variation during the scan, due to different angular mirror positions, we have performed eigenvalue decomposition of the reconstructed probe modes of a Siemens star scan. The resulting mode distribution shows that the dominant eigenmode accounts for 78 % of the total eigenvalue weight, while the remaining modes contribute only minor fractions. After normalization, the single-mode probe reproduces the same spatial profile as the dominant mode in the multi-mode reconstruction. From the object side, reconstructions obtained with single-mode and 4-mode probes exhibit comparable resolution. Therefore, considering the strong dominance of the first eigenmode and the negligible improvement from higher-order modes, we use the single-mode probe reconstructions in our analysis for both accuracy and computational efficiency.

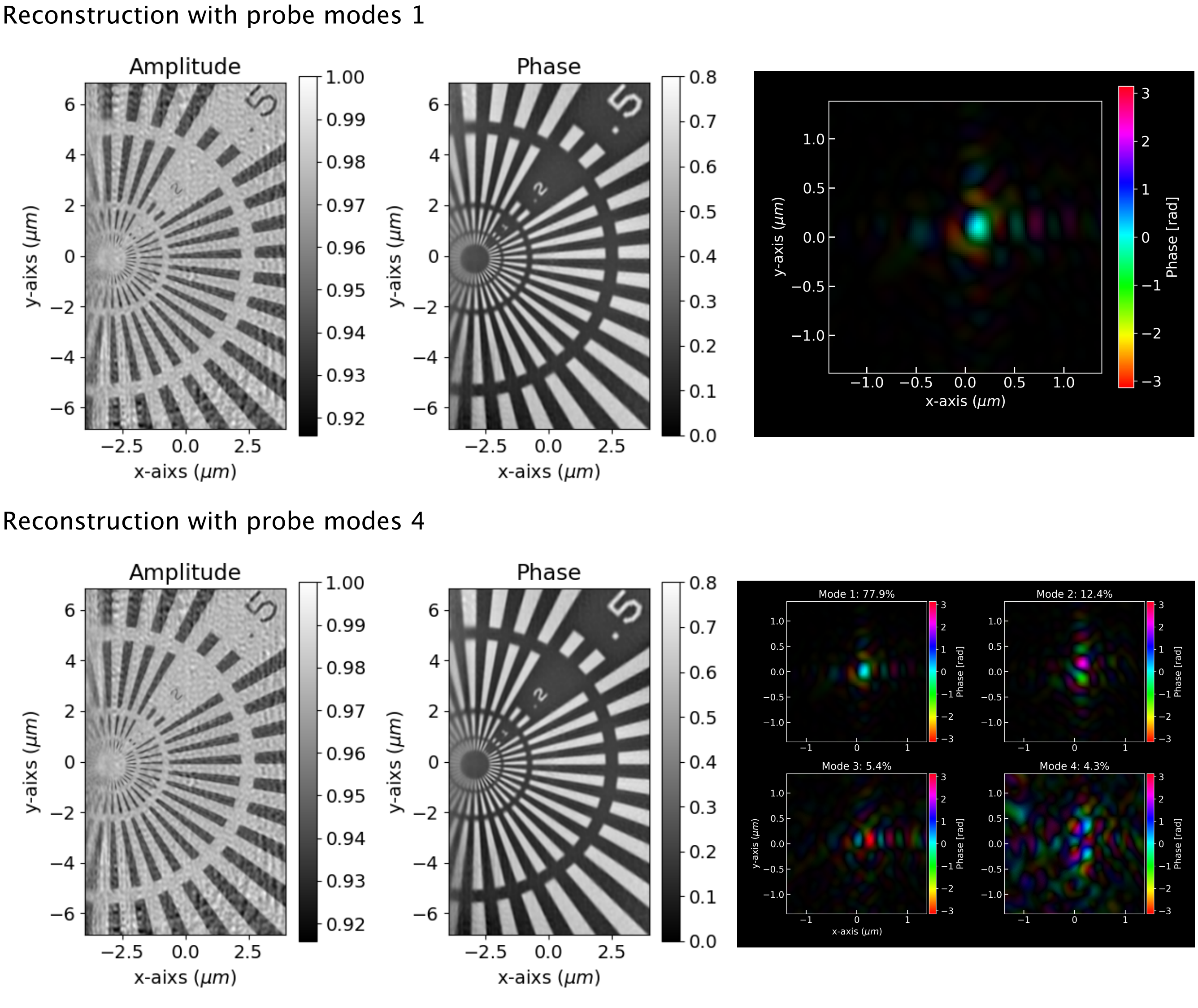

Fig. S1. Reconstruction Comparison with/without probe modes

Resolution Analysis using FRC

In addition to line profile analysis, we performed Fourier ring correlation (FRC) to independently confirm the resolution or mirror scan. The scanning mirror dataset was split into two subsets, one containing diffraction patterns with even indices and the other with odd indices. Taking into account the beam size on the sample (> 200 nm FWHM) and integration interval of 96 nm we are certain that we still fulfill the probe sampling criterion. Each subset was reconstructed using 500 iterations of the difference map algorithm followed by 2500 iterations of maximum likelihood (ML). After reconstruction, the central region on

the Siemens star was cropped for FRC analysis, as shown in Fig. S2 a), which demonstrates consistent recovery of the sample center. The resulting FRC curves (Fig. S2 b) yield a resolution of 34.7 nm according to the half-bit criterion and 36.4 nm according to the one-bit criterion.

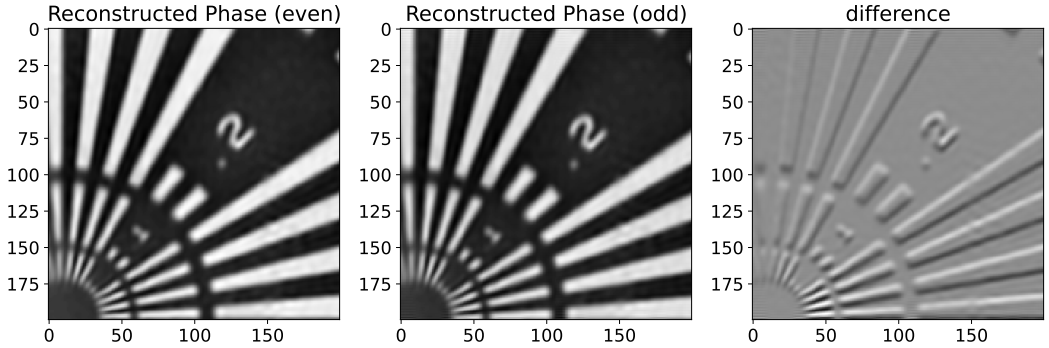

(a) Reconstructed phase of a center of Siemens star from even and odd diffraction patterns.

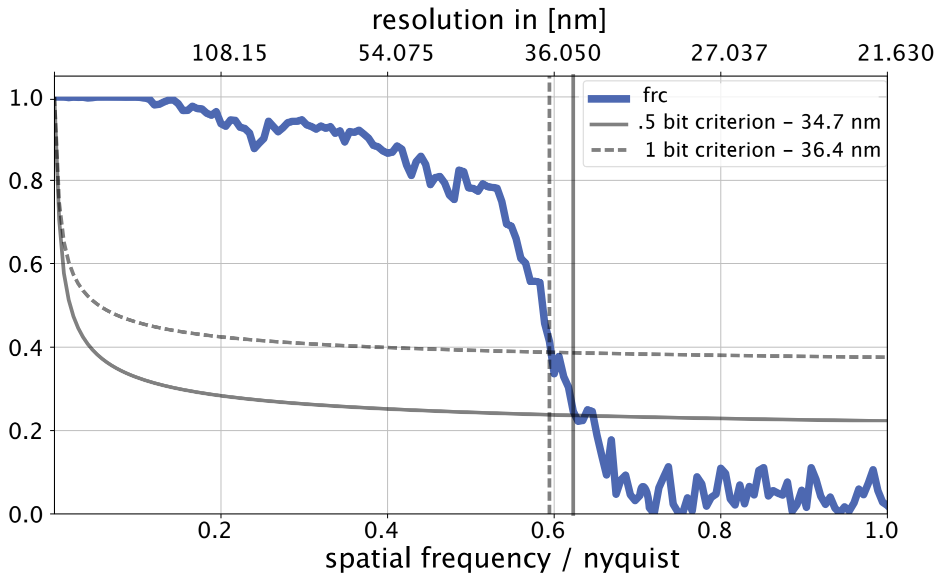

(b) Fourier ring correlation plot, yielding a resolution of 34.7 nm (half-bit) and 36.4 nm (one-bit).

Fig. S2. FRC analysis of reconstructed phase of scanning mirror dataset.

We further validated the resolution by performing FRC of step-scan and fly-scan datasets. Each dataset was reconstructed using 2000 iterations of the extended ptychographic iterative engine followed by 2500 iterations of ML. For FRC analysis, the same central region of the Siemens star was cropped after reconstruction [Fig. S3a], demonstrating consistent recovery of the sample center. The resulting FRC curves [Fig. S3b] indicate resolutions of 27.7 nm (half-bit criterion) and 30.1 nm (one-bit criterion).

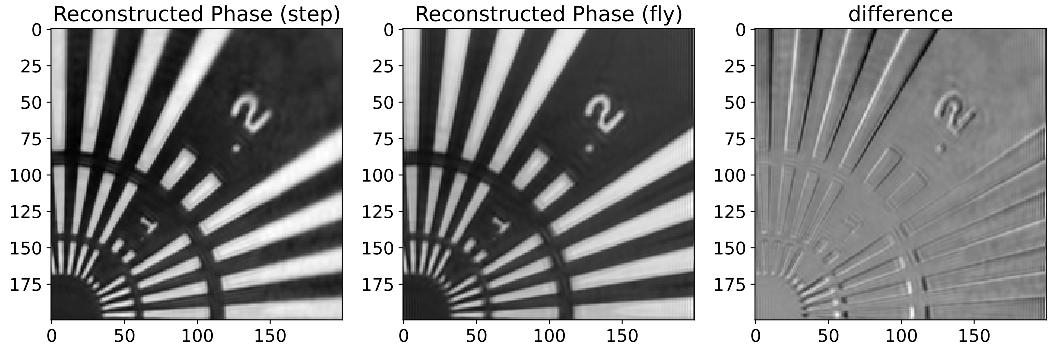

(a) Reconstructed phase of a center of Siemens star from step and fly scan diffraction patterns.

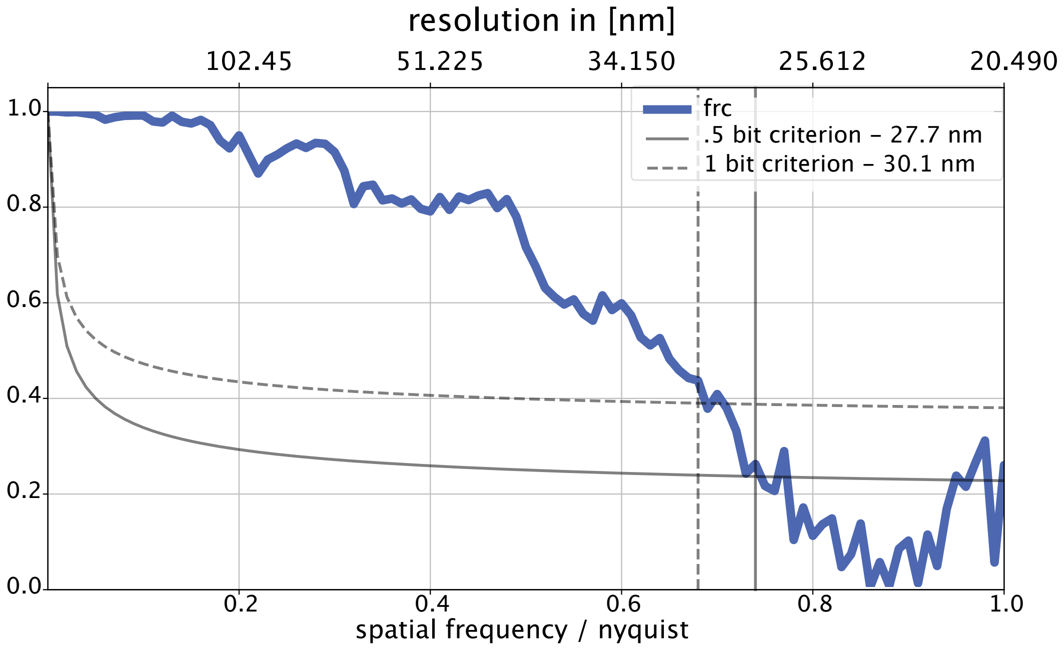

(b) Fourier ring correlation plot, yielding a resolution of 27.7 nm (half-bit) and 30.1 nm (one-bit).

Fig. S3. FRC analysis of reconstructed phase of step scan and fly scan datasets.
